# Periodic Limb Movements during Sleep: A New Sleep-Related Cardiovascular Risk Factor?

**DOI:** 10.3389/fneur.2013.00116

**Published:** 2013-08-12

**Authors:** Maria Alessandria, Federica Provini

**Affiliations:** ^1^Department of Biomedical and NeuroMotor Sciences, Bologna University, Bologna, Italy; ^2^IRCCS Istituto delle Scienze Neurologiche, Bologna, Italy

**Keywords:** periodic limb movements during sleep, hypertension, cardiovascular risk, cerebrovascular risk, stroke, restless legs syndrome

## Abstract

In recent years, a growing body of evidence suggests that periodic limb movements during sleep (PLMS) are associated with hypertension, cardiovascular, and cerebrovascular risk. However, several non-mutually exclusive mechanisms may determine a higher cardiovascular risk in patients with PLMS and the link between the two remains controversial. Prospective data are scant and the temporal relationship between PLMS and acute vascular events is difficult to ascertain because although PLMS may lead to acute vascular events such as stroke, stroke may also give rise to PLMS. This article describes the clinical and polygraphic features of PLMS and examines the literature evidence linking PLMS with an increased risk for the development and progression of cardiovascular diseases, discussing the possible pathways of this association.

## Historical Note

In 1953, Symonds used the term “nocturnal myoclonus” (NM) to describe a series of different motor phenomena occurring during sleep and relaxed wakefulness, sharing the common feature of muscular contractions of the extremities. In the absence of polygraphic recordings, Symonds postulated an epileptic origin of the jerks (1). In the mid-1960s, Lugaresi and colleagues first recorded NM polygraphically in patients with restless legs syndrome (RLS) and other neurological diseases, publishing a series of studies describing NM electroencephalographic (EEG) and electromyographic (EMG) correlates (2, 3). In 1980, Coleman and coworkers suggested the term “periodic movements in sleep” because the muscular contractions characterizing NM are not truly myoclonic and usually have muscle potentials longer than those characterizing myoclonus (less than 250 ms) (4). The term “periodic leg movements in sleep” and the latest term “periodic limb movements during sleep” (PLMS) used by the International Classification of Sleep Disorders (ICSD-II) emphasize the observation that PLMS are usually present in the legs, but that the arms may be involved as well (5, 6). The term “periodic limb movement disorder” (PLMD) refers to patients with periodic limb movements occurring at a rate of more than 5/h in children and more than 15/h in adults (PLM Index, PLMI), and clinical sleep disturbance that cannot be explained by another sleep disorder (6).

## Clinical Features

**Figure 1:**
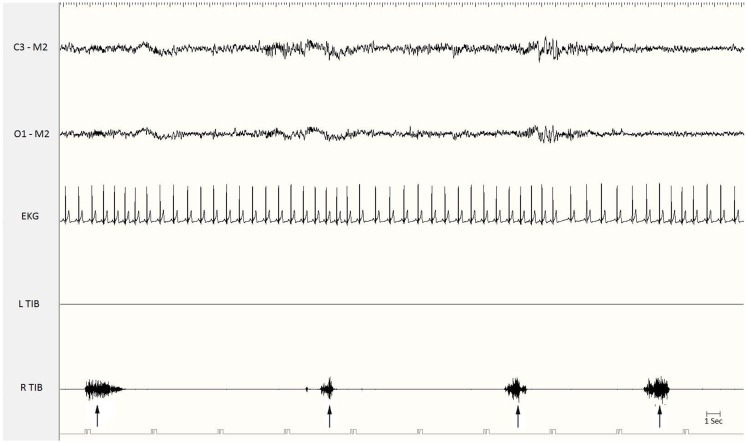
**Polysomnographic recording of periodic limb movements during stage 2 non-REM sleep**. Electromyographic bursts on the right anterior tibialis muscle (R. Tib) recur periodically every 15–20 s (see the arrows). EKG, electrocardiogram; Tib, tibialis anterior; R, right; L, left.

Periodic limb movements during sleep are spontaneous sleep-related movements, frequently involving flexion of the toe, ankle, knee, and hip. Most patients experience repeated flexion of the lower extremities, but some also complain of arm movements. Each movement lasts 0.5–10 s and occurs at intervals of 5–90 s, with a remarkable periodicity of approximately 20–40 s (Figure 1). PLMS are most frequent during non-rapid eye movement (NREM) sleep stages 1 and 2. The movements become less frequent during stage 3 of NREM sleep and during REM sleep. Periodic limb movements may occur during quiet wakefulness, before sleep onset, or in the course of nocturnal waking episodes: during wakefulness the movements have been called periodic limb movements while awake (PLMW) (6). PLMS have been associated with several medical conditions, but are especially frequent in RLS. PLMS may be also an isolated phenomenon, common in healthy adults, without any clinical relevance. PLMS may begin at any age, but they appear to be relatively uncommon until age 40 and then increase with age, with an estimated prevalence of 5–6% in the normal population 30–49 years of age, and 30% or more in people 50 years of age or older (7).

## Polysomnographic Features

**Figure 2:**
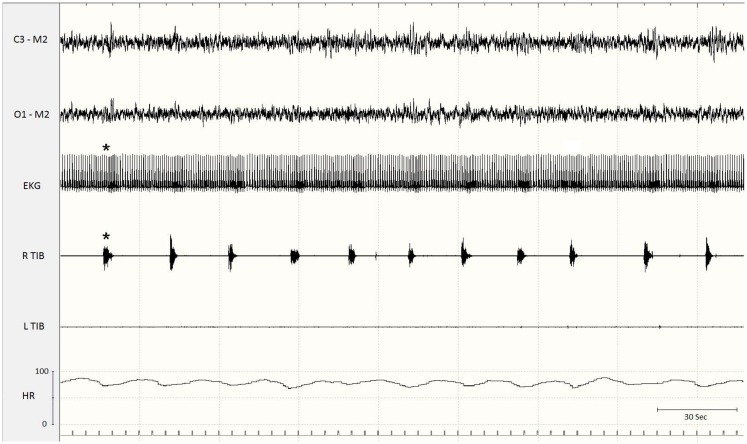
**Excerpt of a polysomnographic tracing at a low paper speed**. Note the association of periodic limb movements with increases in heart rate (asterisks). EKG, electrocardiogram; Tib, tibialis anterior; R, right; L, left; HR, heart rate.

The presence of PLMS is evaluated by polysomnographic (PSG) recordings using surface electrodes placed over both anterior tibialis muscles. Currently accepted methods for recording and scoring PLMS, revised by the World Association of Sleep Medicine in collaboration with a task force from the International RLS Study Group (8) and by a task force of the American Academy of Sleep Medicine (9), are based on the amplitude of tibialis anterior EMG. Each event starts when the EMG amplitude exceeds 8 mV above baseline and ends when the amplitude remains below 2 mV above baseline for at least 0.5 s. The movements must be 0.5–10 s in duration. A sequence of four or more such movements during any sleep stage separated by an interval of at least 5 s and not more than 90 s is considered PLMS (8). However, PSG is an expensive investigation, and alternative methods, such as actigraphic techniques and portable low-cost movement sensor devices have been proposed, particularly for community studies and for automatic detection of PLMS in ambulatory patients (10, 11). Actigraphy may be considered a practical and reliable tool and has been validated against PSG (12, 13). PLMS may be associated with no changes on the EEG and no other evidence of arousal or may instead lead to partial or full arousals. Moreover, the time relationship of the EEG arousals with the leg movements varies: arousals or microarousals (MA) can follow, precede, or accompany the limb movements, suggesting that arousals are not simply the consequence of PLMS and that EEG arousals and PLMS may be separate expressions of a common mechanism (14–17). On the basis of PSG findings, many studies have found no or minimal correlations between PLMS and objective or subjective measures of sleep-wake disturbances, suggesting that PLMS are not responsible for sleep impairment, but often represent only an incidental PSG observation (17, 18). By contrast, some clinicians suggest that PLMS may be an important cause of sleep complaints and that their clinical relevance is related to the EEG signs of arousal they produce: when no arousals are present, patients have no sleep-wake disturbances (4, 19). In any case, it is increasingly clear that PLMS may be associated with pure autonomic activation alone, in the absence of an EEG arousal. Spectral EEG and heart rate (HR) analyses at the time of PLMS in patients with RLS and/or PLMD revealed a variety of complex variations in cortical activity and HR associated with the PLMS, including an increase in HR (Figure 2) and delta band EEG activity before the leg movements, independent of MA (17, 20, 21).

## Pathogenesis and Pathophysiology

The exact origin and pathophysiology of the PLMS remain unclear. A cortical origin is unlikely because electrophysiologic studies demonstrated the lack of any cortical potential preceding the PLMS (22–24). A subcortical origin has been suggested on the basis of the PLMS being synchronous with rhythmic fluctuations of EEG activity and with brainstem-generated autonomic rhythms such as HR and blood pressure (BP) (25, 26). Functional magnetic resonance imaging studies in patients with PLMS and RLS showed an involvement of the red nucleus and brainstem and no apparent cortical activation (27). Neurophysiologic studies support the hypothesis that the underlying mechanisms are active at or rostral to the pontine level (28). Because of their resemblance to the Babinski sign, some authors attribute the PLMS to suppression of supraspinal descending inhibitory pathways on the pyramidal tract (29). The observation that PLMS may be present below a complete spinal cord lesion indicates that PLMS can be directly generated in the spinal cord (24, 30–32). No propriospinal pattern of propagation was found in electrophysiologic studies, showing that the leg muscles are the most frequently involved in PLMS with any caudal or rostral propagation typical of propriospinal myoclonus (24, 33). Muscle activation did not show a consistent recruitment pattern from one PLMS to another, indicating the engagement of different, independent, and sometimes unsynchronized generators for each PLMS. These data suggested an abnormal hyperexcitability along the entire spinal cord, especially in lumbo-sacral and cervical segments, as a primary cause of PLMS, triggered by state-dependent sleep-related factors located at a supraspinal level (24). On the other hand, different studies implicated the peripheral nervous system in the pathogenesis of PLMS, but it is still an unresolved question whether peripheral dysfunction triggers PLMS or is simply coincidental. The efficacy of l-DOPA, dopamine agonists, and opioids in reducing PLMS suggests an involvement of either the dopaminergic or the opioid system in their pathogenesis. The association of PLMS with RLS, narcolepsy, and REM sleep behavior disorder reinforces the possibility of an impaired central dopaminergic transmission (17). Finally, in view of the role of iron in the origin of RLS, it was observed that patients with RLS and low serum ferritin have higher PLMS indexes. Although there is some evidence that magnesium deficiency may play a role in the development of RLS, the role of this deficit in the pathogenesis of PLMS has not been determined (34).

## Are PLMS Associated with Cardio and Cerebrovascular Disease?

The sympathetic hyperactivity associated with PLMS has been hypothesized to engender cardio- and cerebrovascular comorbidities (35). PLMS, either in association with RLS or as an isolated phenomenon, have been related to autonomic activations. PLMS are associated with a significant transient increase in BP and HR, particularly pronounced when PLMS are accompanied by MA, but even present in the absence of EEG arousal changes (20, 36). In NREM sleep, the primary cardiac effect of the PLMS consists in a sharp increase in sympathetic activation associated with a low reduction in parasympathetic tonus, as reflected by fluctuations in all time and frequency HR variability (HRV) measures (37). EEG and HR changes may start some seconds before the beginning of PLMS (38). Cardiac activation is significantly greater when leg movements are associated with the end of obstructive sleep apnea episodes, compared to respiratory events not associated with leg movements (39). The amplitude of the HR changes associated with PLMS varies with age. HR changes are more pronounced in bilateral PLMS are than in unilateral movements (38). The repetitive abnormal HR rises connected with PLMS might have a long-term negative effect on the cardiovascular system correlated positively with the PLMS index (40). Pennestri and coworkers, monitoring HRV and BP in a group of ten RLS patients, demonstrated that PLMS are associated with a significant increase in HR and diastolic and systolic arterial pressure, whether or not movements are associated with MA; BP changes in PLMS associated with MA were greater than those in PLMS not associated with MA. Moreover, BP changes increased with age and disease duration: the authors concluded that PLMS could contribute to the risk of cardiovascular diseases in patients with RLS, especially in the elderly (41). The same group recently demonstrated that PLMS were associated with sudden and significant increases in HR and BP in both RLS patients and healthy subjects, but cardiovascular increases were more pronounced in RLS patients than in healthy subjects (42). Treatment with low dose pramipexole decreased the number of PLMS and normalized the increased PLMS-related HR response in RLS patients, controlling abnormal autonomic activation, without effects on tonic vegetative regulation (40). Recurring BP and HR rises may increase the risk of cardiovascular diseases by promoting arterial oxidative stress and inflammation which are well-known phenomena involved in the pathogenetic mechanism of atherosclerosis (40, 43). Systemic inflammation may be measured with a variety of markers, including C-reactive protein (CRP), an acute-phase response protein implicated in a broad range of cardiovascular diseases. CRP elevation in association with PLMS in RLS patients has been described (44). Another study found that levels of CRP and lipoprotein-associated phospholipase A2 (Lp-PLA2), a marker of vulnerable plaque prone to rupture, predict both cardiovascular and cerebrovascular events, and were significantly increased in patients with elevated PLMSI and positively correlated with the index (45). Large epidemiological surveys have found a significant comorbidity between RLS and cardiovascular diseases (46, 47). In a group of 23 patients with congestive heart failure (CHF) and Cheyne–Stokes respiration, 52% of patients had a PLMS index>25 (versus 11% of controls) and one-third of CHF patients had a PLMS index>50 (48). Two successive studies demonstrated an increased PLMS index in CHF patients (49, 50). A single case report described a CHF patient with PLMS who recovered after heart transplantation (51). In contrast with this experience, Javaheri and colleagues reported that after heart transplantation one-third of patients showed a PLMS index>15 (52), independent of sleep-disordered breathing. In summary, PLMS are frequently observed in CHF patients, also after cardiac transplantation (35).

The relationship between PLMS and hypertension remains unclear although some studies reported an association between chronic elevations of BP and PLMS (53, 54). Eighteen percent of 91 subjects with hypertension had PLMS, and the prevalence of PLMS was directly proportional to the severity of hypertension (53). Billars and coworkers studied the association between hypertension and PLMS in a group of 861 RLS patients, finding that the risk of hypertension was higher in patients with a PLMS index>30 (54). Other studies did not confirm the association between hypertension and PLMS. Ohayon and Roth examined RLS and PLMD prevalence in a cross-sectional population study of 18,980 subjects aged ≥15 years in five European countries through a telephone interview: hypertension (treated or untreated) was significantly associated with RLS but not PLMS. It should be emphasized that in this study, the diagnosis of PLMS was not made by PSG but by the validated Sleep-EVAL system questionnaire (55). A recent cross-sectional study involving 314 children showed that children with PLMS were at significantly higher risk for nocturnal systolic and diastolic hypertension (56). Non-dipping BP has been associated with increased cardiovascular morbidity, such as hypertensive organ damage and greater left ventricular mass index, even in the absence of sustained diurnal or nocturnal hypertension (57).

In patients with renal failure, PLMS is associated independently with increased estimated cardiovascular and cerebrovascular risk (58). In hemodialysis patients with RLS, severe PLMS has been associated with further left ventricular structure abnormalities (59). PLMS in both end-stage renal disease and systolic heart failure has been shown to increase the mortality risk (60, 61). A recent study found that PLMS frequency is associated with incident cardiovascular disease in a community-based elderly male population (62).

There is anecdotal evidence that stroke may lead directly to RLS/PLMS (35). The opposite relationship is also possible since PLMS with associated autonomic activation may be a risk factor for stroke. Coelho et al., retrospectively studying 40 patients with a history of stroke and 40 control patients matched for age, sex, and risk factors, found a greater prevalence and severity of PLMS in patients with a history of stroke than in controls (63). Prospective studies are needed to confirm these findings in order to establish a possible association between RLS/PLMS and stoke.

Finally, PLMS may represent a possibly and scarcely evaluated vascular risk factor in some neurodegenerative diseases frequently associated with sudden death such as myotonic dystrophy type 1 (64).

## Conclusion and Further Directions

The literature suggests that PLM patients are at increased risk of developing hypertension, cardiovascular, and cerebrovascular disease, but inconsistencies persist (35, 65). Additional prospective studies are needed to confirm this association and to clarify both the long and short-term effects of dopaminergic drugs on PLMS-associated BP and HR changes, and consequently to understand the impact of PLMS treatment on hypertension, stroke, and heart disease prevention.

## Conflict of Interest Statement

The authors declare that the research was conducted in the absence of any commercial or financial relationships that could be construed as a potential conflict of interest.

## References

[1] Symonds CP Nocturnal myoclonus. J Neurol Neurosurg Psychiatry (1953) 16:166–71 10.1136/jnnp.16.3.166 13085198 PMC503132

[2] Lugaresi E Tassinari CA Coccagna G Ambrosetto C Particularite’s cliniques et polygraphiques du syndrome d’impatience des membres inferieurs. Rev Neurol (1965) 113:545–55

[3] Lugaresi E Coccagna G Gambi D Berti Ceroni G Poppi M A propos de quelques manifestations nocturnes myocloniques (nocturnal myoclonus de Symonds). Rev Neurol (1966) 115:547–55 5971319

[4] Coleman RM Pollak CP Weitzman ED Periodic movements in sleep (nocturnal myoclonus): relation to sleep disorders. Ann Neurol (1980) 8:416–21 10.1002/ana.410080413 7436384

[5] Chabli A Michaud M Montplaisir J Periodic arm movements in patients with the restless legs syndrome. Eur Neurol (2000) 44:133–8 10.1159/000008221 11053959

[6] American Academy of Sleep Medicine International Classification of Sleep Disorders, Diagnostic & Coding Manual. 2nd ed. Westchester, IL: American Academy of Sleep Medicine (2005).

[7] Bixler EO Kales A Vela-Bueno A Jacoby JA Scarone S Soldatos CR Nocturnal myoclonus and nocturnal myoclonic activity in a normal population. Res Commun Chem Pathol Pharmacol (1982) 36:129–40 7079579

[8] Zucconi M Ferri R Allen R Baier PC Bruni O Chokroverty S International Restless Legs Syndrome Study Group (IRLSSG). The official World Association of Sleep Medicine (WASM) standards for recording and scoring periodic leg movements in sleep (PLMS) and wakefulness (PLMW) developed in collaboration with a task force from the International Restless Legs Syndrome Study Group (IRLSSG). Sleep Med (2006) 7(2):175–83 16459136 10.1016/j.sleep.2006.01.001

[9] Iber C Ancoli-Israel S Chesson A Quan SF The AASM Manual for the Scoring of Sleep and Associated Events: Rules, Terminology and Technical Specifications. Westchester, IL: American Academy of Sleep Medicine (2007).

[10] Shochat T Oksenberg A Hadas N Molotsky A Lavie P The KickStrip: a novel testing device for periodic limb movement disorder. Sleep (2003) 26(4):480–3 12841376 10.1093/sleep/26.4.480

[11] Rauhala E Virkkala J Himanen SL Periodic limb movement screening as an additional feature of Emfit sensor in sleep-disordered breathing studies. J Neurosci Methods (2009) 178(1):157–61 10.1016/j.jneumeth.2008.11.019 19100767

[12] Sforza E Johannes M Bassetti C The PAM-RL ambulatory device for detection of periodic leg movements: a validation study. Sleep Med (2005) 6:407–13 10.1016/j.sleep.2005.01.004 16139771

[13] Kemlink D Pretl M Sonka K Nevsimalova S A comparison of polysomnographic and actigraphic evaluation of periodic limb movements in sleep. Neurol Res (2008) 30(3):234–8 10.1179/016164107X229911 17767810

[14] Montplaisir J Boucher S Gosselin A Poirier G Lavigne G Persistence of repetitive EEG arousals (K-alpha complexes) in RLS patients treated with L-DOPA. Sleep (1996) 19:196–9 8723375 10.1093/sleep/19.3.196

[15] Karadeniz D Ondze B Besset A Billiard MEEG. arousals and awakenings in relation with periodic leg movements during sleep. J Sleep Res (2000) 9:273–7 10.1046/j.1365-2869.2000.00202.x 11012867

[16] El-Ad B Chervin RD The case of a missing PLM. Sleep (2000) 23:450–1 10875552

[17] Provini F Vetrugno R Ferri R Montagna P Periodic limb movements in sleep. In: Hening W Allen R Chokroverty S Saunders C editors. Restless Legs Syndrome. Philadelphia: Elsevier Inc. (2009). p. 119–33

[18] Mendelson WB Are periodic leg movements associated with clinical sleep disturbances? Sleep (1996) 19:219–23 8723379 10.1093/sleep/19.3.219

[19] Guilleminault C Raynal D Weitzman ED Dement WC Sleep related periodic myoclonus in patients complaining of insomnia. Trans Am Neurol Assoc (1975) 100:19–21 1226607

[20] Sforza E Jouny C Ibanez V Time-dependent variation in cerebral and autonomic activity during periodic leg movements in sleep: implications for arousal mechanisms. Clin Neurophysiol (2002) 113:883–91 10.1016/S1388-2457(02)00066-4 12048047

[21] Ferrillo F Beelke M Canovaro P Watanabe T Aricò D Rizzo P Changes in cerebral and autonomic activity heralding periodic limb movements in sleep. Sleep Med (2004) 5:407–12 10.1016/j.sleep.2004.01.008 15223001

[22] Lugaresi E Cirignotta F Coccagna G Montagna P Nocturnal myoclonus and restless syndrome. In: Fahn S Marsden CD Van Woert MH editors. Myoclonus: Advances in Neurology. (Vol. 43), New York: Raven Press (1986). p. 295–307 3946114

[23] Trenkwalder C Bucher SF Oertel WH Proeckl D Plendl H Paulus W Bereitschaftspotential in idiopathic and symptomatic restless legs syndrome. Electroencephalogr Clin Neurophysiol (1993) 89:95–103 10.1016/0168-5597(93)90090-C 7683607

[24] Provini F Vetrugno R Meletti S Plazzi G Solieri L Lugaresi E Motor pattern of periodic limb movements during sleep. Neurology (2001) 57(2):300–4 10.1212/WNL.57.2.300 11468316

[25] Lugaresi E Coccagna G Mantovani M Lebrun R Some periodic phenomena arising during drowsiness and sleep in man. Electroencephalogr Clin Neurophysiol (1972) 32:701–5 10.1016/0013-4694(72)90106-X 4121520

[26] Parrino L Boselli M Buccino GP Spaggiari MC Di Giovanni G Terzano MG The cyclic alternating pattern plays a gate-control on periodic limb movements during non-rapid eye movement sleep. J Clin Neurophysiol (1996) 13:314–23 10.1097/00004691-199607000-00005 8858493

[27] Bucher SF Seelos KC Oertel WH Reiser M Trenkwalder C Cerebral generators involved in the pathogenesis of the restless legs syndrome. Ann Neurol (1997) 41:639–45 10.1002/ana.410410513 9153526

[28] Wechsler LR Stakes JW Shahani BT Busis NA Periodic leg movements of sleep (nocturnal myoclonus): an electrophysiological study. Ann Neurol (1986) 19:168–73 10.1002/ana.410190210 3963759

[29] Smith RC The Babinski response and periodic limb movement disorder. J Neuropsychiatry Clin Neurosci (1992) 4:233–4 1627987 10.1176/jnp.4.2.233

[30] Yokota T Hirose K Tanabe H Tsukagoshi H Sleep-related periodic leg movements (nocturnal myoclonus) due to spinal cord lesion. J Neurol Sci (1991) 104:13–8 10.1016/0022-510X(91)90210-X 1919596

[31] Lee MS Choi YC Lee SH Lee SB Sleep-related periodic leg movements associated with spinal cord lesions. Mov Disord (1996) 11:719–22 10.1002/mds.870110619 8914100

[32] De Mello MT Lauro FA Silva AC Tufik S Incidence of periodic leg movements and of the restless legs syndrome during sleep following acute physical activity in spinal cord injury subjects. Spinal Cord (1996) 34:294–6 10.1038/sc.1996.53 8963978

[33] Brown P Thompson PD Rothwell JC Day BL Marsden CD Axial myoclonus of propriospinal origin. Brain (1991) 114:197–214 1998882

[34] Popoviciu L Asgian B Delast-Popoviciu D Alexandrescu A Petrutiu S Bagathal I Clinical, EEG, electromyographic and polysomnographic studies in restless legs syndrome caused by magnesium deficiency. Rom J Neurol Psychiatry (1993) 31:55–61 8363978

[35] Walters AS Rye DB Review of the relationship of restless legs syndrome and periodic limb movements in sleep to hypertension, heart disease, and stroke. Sleep (2009) 32(5):589–97 19480225 10.1093/sleep/32.5.589 PMC2675893

[36] Winkelman JW The evoked heart rate response to periodic leg movements of sleep. Sleep (1999) 22(5):575–80 10450592 10.1093/sleep/22.5.575

[37] Sforza E Pichot V Barthelemy JC Haba-Rubio J Roche F Cardiovascular variability during periodic leg movements: a spectral analysis approach. Clin Neurophysiol (2005) 116:1096–104 10.1016/j.clinph.2004.12.018 15826850

[38] Ferri R Zucconi M Rundo F Spruyt K Manconi M Ferini-Strambi L Heart rate and spectral EEG changes accompanying periodic and non-periodic leg movements during sleep. Clin Neurophysiol (2007) 118:438–48 10.1016/j.clinph.2006.10.007 17140849

[39] Yang CK Jordan AS White DP Winkelman JW Heart rate response to respiratory events with or without leg movements. Sleep (2006) 29:553–6 16676789 10.1093/sleep/29.4.553

[40] Manconi M Ferri R Zucconi M Clemens S Rundo F Oldani A Effects of acute dopamine-agonist treatment in restless legs syndrome on heart rate variability during sleep. Sleep Med (2011) 12(1):47–55 10.1016/j.sleep.2010.03.019 20851046

[41] Pennestri MH Montplaisir J Colombo R Lavigne G Lanfranchi PA Nocturnal blood pressure changes in patients with restless legs syndrome. Neurology (2007) 68:1213–8 10.1212/01.wnl.0000259036.89411.52 17420405

[42] Pennestri MH Montplaisir J Fradette L Lavigne G Colombo R Lanfranchi PA Blood pressure changes associated with periodic leg movements during sleep in healthy subjects. Sleep Med (2013) 14(6):555–61 10.1016/j.sleep.2013.02.005 23643655

[43] Bonomini F Tengattini S Fabiano A Bianchi R Mezzani R Atherosclerosis and oxidative stress. Histol Histopathol (2008) 23:381–90 18072094 10.14670/HH-23.381

[44] Trotti LM Rye DB De Staercke C Hooper WC Quyyumi A Bliwise DL Elevated C-reactive protein is associated with severe periodic leg movements of sleep in patients with restless legs syndrome. Brain Behav Immun (2012) 26(8):1239–43 10.1016/j.bbi.2012.06.003 22750520 PMC3468666

[45] Bekci TT Kayrak M Kiyici A Ari H Teke T Maden E The relation between Lp-PLA2 levels with periodic limb movements. Sleep Breath (2012) 16:117–22 10.1007/s11325-010-0464-y 21221825

[46] Phillips B Hening W Britz P Mannino D Prevalence and correlates of restless legs syndrome: results from the 2005 national sleep foundation poll. Chest (2006) 129:76–80 10.1378/chest.129.1.76 16424415

[47] Winkelman JW Finn L Young T Prevalence and correlates of restless legs syndrome symptoms in the Wisconsin sleep cohort. Sleep Med (2006) 7:545–52 10.1016/j.sleep.2006.01.004 16740407

[48] Hanly PJ Zuberi-Khokhar N Periodic limb movements during sleep in patients with congestive heart failure. Chest (1996) 109:1497–502 10.1378/chest.109.6.1497 8769500

[49] Javaheri S Sleep disorders in systolic heart failure: a prospective study of 100 male patients. The final report. Int J Cardiol (2006) 106:21–8 10.1016/j.ijcard.2004.12.068 16321661

[50] Skomro R Silva R Alves R Figueiredo A Lorenzi-Filho G The prevalence and significance of periodic leg movements during sleep in patients with congestive heart failure. Sleep Breath (2009) 13:43–7 10.1007/s11325-008-0207-5 18592284

[51] Hanly P Zuberi N Periodic leg movements during sleep before and after heart transplantation. Sleep (1992) 15:489–92 1475562

[52] Javaheri S Abraham WT Brown C Nishiyama H Giesting R Wagoner LE Prevalence of obstructive sleep apnea and periodic limb movement in 45 subjects with heart transplantation. Eur Heart J (2004) 25:260–6 10.1016/j.ehj.2003.10.032 14972428

[53] Espinar-Sierra J Vela-Bueno A Luque-Otero M Periodic leg movements in sleep in essential hypertension. Psychiatry Clin Neurosci (1997) 51:103–7 10.1111/j.1440-1819.1997.tb02370.x 9225372

[54] Billars L Hicks A Bliwise D Hypertension risk and PLMS in restless legs syndrome. Sleep (2007) 30:A297–8

[55] Ohayon MM Roth T Prevalence of restless legs syndrome and periodic limb movement disorder in the general population. J Psychosom Res (2002) 53:547–54 10.1016/S0022-3999(02)00443-9 12127170

[56] Wing YK Zhang J Ho CK Au CT Li AM Periodic limb movement during sleep is associated with nocturnal hypertension in children. Sleep (2010) 33:759–65 20550016 10.1093/sleep/33.6.759 PMC2881712

[57] Hoshide S Kario K Hoshide Y Umeda Y Hashimoto T Kunii O Associations between nondipping of nocturnal blood pressure decrease and cardiovascular target organ damage in strictly selected community-dwelling normotensives. Am J Hypertens (2003) 16:434–8 10.1016/S0895-7061(03)00567-3 12799090

[58] Lindner A Fornadi K Lazar AS Czira ME Dunai A Zoller R Periodic limb movements in sleep are associated with stroke and cardiovascular risk factors in patients with renal failure. J Sleep Res (2012) 21(3):297–307 10.1111/j.1365-2869.2011.00956.x 21917047

[59] Hadjigeorgiou GM George KP Gourgoulianis K Koutedakis Y Stefanidis I Sakkas GK Periodic limb movements in sleep contribute to further cardiac structure abnormalities in hemodialysis patients with restless legs syndrome. J Clin Sleep Med (2013) 9(2):147–53 10.5664/jcsm.2412 23372468 PMC3544383

[60] Benz RL Pressman MR Hovick ET Peterson DD Potential novel predictors of mortality in end-stage renal disease patients with sleep disorders. Am J Kidney Dis (2000) 35:1052–60 10.1016/S0272-6386(00)70039-4 10845816

[61] Yumino D Wang H Floras JS Newton GE Mak S Ruttanaumpawan P Relation of periodic leg movements during sleep and mortality in patients with systolic heart failure. Am J Cardiol (2011) 107:447–51 10.1016/j.amjcard.2010.09.037 21257013

[62] Koo BB Blackwell T Ancoli-Israel S Stone KL Stefanick ML Redline S Osteoporotic Fractures in Men (MrOS) Study Group. Association of incident cardiovascular disease with periodic limb movements during sleep in older men: outcomes of sleep disorders in older men (MrOS) study. Circulation (2011) 124(11):1223–31 10.1161/CIRCULATIONAHA.111.038968 21859975 PMC3265562

[63] Coelho FM Georgsson H Narayansingh M Swartz RH Murray BJ Higher prevalence of periodic limb movements of sleep in patients with history of stroke. J Clin Sleep Med (2010) 6(5):428–30 20957841 PMC2952744

[64] Romigi A Izzi F Pisani V Placidi F Pisani LR Marciani MG Sleep disorders in adult-onset myotonic dystrophy type 1: a controlled polysomnographic study. Eur J Neurol (2011) 18:1139–45 10.1111/j.1468-1331.2011.03352.x 21338442

[65] Provini F Restless legs syndrome, periodic limb movements in sleep, and vascular risk factors. In: Culebras A editor. Sleep, Stroke and Cardiovascular Disease. New York: Cambridge University Press (2013). p. 139–50

